# Effect of storage age and containers on the physicochemical degradation of guggul oleo-resin

**DOI:** 10.1038/s41598-023-39594-5

**Published:** 2023-08-07

**Authors:** Moni Thomas, Niraj Tripathi, Shibu M. Eappen, Kailash C. Meena, Atul Shrivastava, Niranjan Prasad

**Affiliations:** 1 Directorate of Research Services, Jawaharlal Nehru Krishi Vishwa Vidyalaya, Jabalpur, 482004 India; 2 Sophisticated Test and Instrumentation Centre, Cochin University of Science and Technology, Ernakulam, 682022 India; 3 Indian Institute of Natural Resins and Gums, Namkum, Ranchi, 834010 India

**Keywords:** Biochemistry, Biological techniques, Plant sciences

## Abstract

Guggul is a gum oleo-resin, tapped from a data deficient plant- *Commiphora wightii* (Arnott.) Bhandari in India. It is extensively used in ayurvedic drugs and formulations since ages. Natural plant-based products; especially aromatic ones like guggul gum oleo-resin deteriorates, qualitatively during its storage and transits before reaching the industry for its value addition. This economical and ecological loss can be avoided if it is stored in proper containers. Physico-chemical degradation of guggul samples stored were analysed by scanned electron microscopy, fourier transformed infra red, thermogravimatric, Powdered X-ray diffraction and elemental analysis for carbon, hydrogen, nitrogen and sulphur. Physico-chemical degradation of guggul oleo-resin occurs with the age of storage and the type of storage containers used. Among the four storage containers (earthen pot, plastic jar, polythene bag, jute bag) evaluated, earthen pot was found to be the best in checking the qualitative loss of guggul even upto 24 months. The qualitative information generated in the study on guggul storage may be useful to the drug industry and guggul traders. It may encourage them practice storing guggul in earthen pots against current practice of using jute bags and polythene bags, to store it.

## Introduction

Across the continents, natural products are collected, traded, stored and transported before their processing^1^. Unlike synthetic products, natural products are less stable (physically or chemically), depending on its post harvest handling^2^. Degradation or deterioration of natural products^3^ is not only an economical loss^4^ but also a biological as well as ecological loss^5,6^, in a broader term. Conditions and containers used^7^ are crucial for storage of natural resin and gums (NRG). In the process of crushing, sieving and heating of natural products, decomposition takes place^8^. Temperature for initiation of decomposition (TID) or complete decomposition (TCD) of NRG depends on its storage containers and conditions^9^.

Guggul gum is an important gum oleo-resin tapped from data deficient *Commiphora wightii*. The domestic supply is just 10 metric tonnes against the annual industrial demand of 10,000 metric tonnes in India^10,11^. This high value aromatic product tapped and collected from the field reaches industry not earlier than 24 months, through a complex labyrinth trade network. During this transit period, qualitative deterioration^7,12^ cannot be ruled out. Good practices followed for production and collection are important in the primary stages of NRG, but the secondary stage (transport and storage) is of utmost industrial importance for maintaining its bioactive compounds^7^. Guggul gum oleo-resin is used in drug, cosmetic formulations as well as other products^11^. Product development involves processing^13^, mixing of various ingredients^14^ and even heating. Combination index^15^ in drug formulation is vital for effectiveness and stability^16,17^. Quality deterioration of any natural product, including guggul during storage and transit is an important point for consideration^18^, on account of the loss of aromatic oils and bio-active compounds^7^. A qualitatively degraded raw material will differ chemically and physically from its parent material. These physico-chemical changes may also influence its interaction with other materials viz. additive^19,20^, antagonism^21^, potentiation^22^ and synergism^23^ of the drug prepared.

Raw natural products intended for pharmaceutical applications are expected to be qualitatively superior. To maintain the superiority of raw materials it is important to store them in a suitable container. Earthen pot is made up of small soil particles and before its use it is baked. Earthen pots are preferred because of their low cost and easy construction followed by low-temperature variation that keeps the stored material cool^24^. Quality of plastic is important during selection of plastic jar as a container for herbal products. The raw materials stored in the plastic jar may lose its quality due to exposure of light and air. Plastic containers have varying degree of permeability and oxygen inside the container may degrade the aroma. Phthalate contamination in herbal products stored in plastic containers is also reported. Phthalate is a compound found in the plastic and has adverse effect on human health^25^. In order to address this vital issue, the physio-chemical analysis of guggul stored in different containers and for various durations was conducted through Scanning electron microscopy (SEM), Fourier transform infrared (FT-IR) spectroscopy, Thermogravimetry analysis (TGA) techniques as well as Powder X-ray diffraction (PXRD) analysis. These techniques can be applied to determine the textural and structural changes of stored guggul or after its transits^26,27^. SEM determines surface morphology and confirms sample fragmentation during storage. FT-IR spectroscopy, on the other hand, establishes the presence or absence of certain functional groups as well as the mineralization process^27,28^. TGA provides comprehensive information on the mass reduction and breakdown of natural compounds at various temperatures^26^.

## Materials and methods

Guggul gum yielding plants (*C. wightii*) is found along the Chambal ravines of Morena district of Madhya Pradesh, India. *C. wightii* plant was identified by Dr. Gyanendra Tiwari (Taxonomist), Department of Plant Physiology, Jawaharlal Nehru Krishi Vishwa Vidyalaya, Jabalpur. The plant species was also identified and confirmed by using DNA barcoding primers. Fresh guggul gum was collected in the month of May during the year 2016 directly from the local collectors. Freshly collected guggul gum was brought to the laboratory. The 12 kg guggul was divided into 48 parts, each consisting of 250 g. Four containers viz. Earthen pot (T_1_), Plastic jar (T_2_), Polythene bag (T_3_) and Jute bag (T_4_) were used to store fresh guggul gum. Each of the 4 containers had 12 pieces. All the containers filled with 250 g of guggul gum were stored at room temperature in the laboratory 12 and 24 months. These were then subjected to physico-chemical and qualitative analysis at the Sophisticated Analytical Instrument Facility (STIC**)**, Kochi, Kerala, India. The present work was carried out under the ICAR funded All India Network Project on Harvesting, Processing and Value Addition of Natural Resins and Gums. Guggul (*C. wightii*) is allotted as a mandatory crop for our institute. The collection of plant material, complied with relevant institutional, national, and international guidelines and legislation.

### CHNS analysis

Fresh and stored guggul samples weighing 5–10 mg from different containers were separately wrapped in silver foil in the form of a capsule. This was placed in the auto sampler. The silver foil capsule containing the sample was dropped into the reactor chamber, priorily flushed with excess oxygen. On complete oxidation in presence of tungsten trioxide at about 1150 °C, the resultant gaseous products consisted of CO_2_, H_2_ONO_x_ and SO_2_. At 850 °C, when this gaseous mixture was allowed to pass through a silica tube, packed with copper granules, excess oxygen binds and reduces them. The gas leaving the silica tube now consisting of CO_2_, H_2_O, N_2_ and SO_2,_ were adsorbed at appropriate traps. The carrier gas was high purity helium. The gaseous mixture was then passed through a gas chromatographic system where the gaseous species were separated and detected using a Thermal Conductivity Detector. By elemental analysis of standard substances—sulfanilic acid, the blank values were taken from the carrier gas run calibration^29^.

### Fourier transform infrared spectroscopy (FT-IR)

Minute quantity (1 mg) of fresh as well as stored samples of guggul were separately mixed with KBr in the ratio of 1:100 approximately (w/w), followed by grinding to fine powder with mortar and pestle for 3–4 min. A pellet was made from the powder by transferring it into 13 mm diameter die and applying 7 ton psi of pressure with a hydraulic press. The resultant fine pellet was subjected to FT-IR analysis using a universal pellet holder. Infrared spectral data were collected on Thermo Avtar 370 FT-IR spectrometer. Spectra were collected over a Broad Band (BB) range varying from 4000 to 400 cm^−1^at 4 cm^−1^ resolutions with an interferogram of 32 scans (BB) following the procedure suggested by Telkapalliwar and Shivankar^30^.

### SEM analysis

Individual guggul sample (fresh and stored) was secured to a brass stub by smearing a small piece of adhesive carbon tape. The sample was then coated with gold using a sputtering unit (model: JFC1600) for 10 s at 10 mA of current. The gold-coated sample was later placed in a SEM chamber (Jeol, JSM 6390LA) and secondary electron images were recorded^31^.

### Thermal Gravimetric Analysis (TGA)

Thermal gravimetric analysis (TGA) was performed using a TGA Q50 Thermo-gravimetric analyzer (TA Instruments, USA) to evaluate sample’s thermal stability and physico-sorbed water evolution. Approximately 5 mg of sample was scanned from ambient (approximately 30 °C) to 800 °C at 10 °C min^−1^ with Al_2_O_3_ as a reference^32^ for TGA.

### X-Ray Diffraction Analysis

Sample (5 mg) of guggul gum (fresh and stored) was smeared or on the low back ground sample holder (amorphous silica holder) and fixed on the sample stage in Goniometer. The instrument was set with Broad Band (BB) geometry, while the current and voltage was set to 40 mV and 35 mA respectively. The data thus obtained was collected using Bruker D8 Advance in the 2 theta range 3 to 80 degree with a step size of 0.02 °C per minute^33^.

## Results and discussion

The term "storage" refers to the holding of commodities in godowns or storage structures. Commodities stored are kept in conditions for a period, or until they are needed for further processing, marketing, or consumption^34^. Storage may be such that the qualitative as well as quantitative characters are retained to the maximum^35^.

### CHNS analysis

Field fresh guggul sample collected had N (3.75%), C (59.54%), H (12.84%) and S (6.88%).

#### After 12 months

Among the twelve months old stored samples (Table 1), the samples stored in jute bag had highest percent of N (3.59%) and H (10.71%) while lowest S (0.75%). The C content was highest (55.77%) in samples stored in polythene bag while it was lowest (48.36%) in plastic jars. Samples stored in earthen pots had highest (6.51%) S content but had lowest H (8.66%). The highest decline (59.47%) in N content was noticed in guggul sample stored in polythene bag while the lowest (4.27%) decline in N was in the sample stored in jute bag. Decline in C content was highest (18.77%) in plastic jar, lowest (6.33%) in polythene bag. Lowest (5.37%) decline in S content was noticed in earthen pot while the highest (89.09%) decline was in jute bag. However the lowest (16.59%) decline in H content was observed in jute bag.

**Table 1:** CHNS of guggul gum oleo-resin stored in different containers.

Storage container	Sample	Percentage of elements			
		N	C	S	H
	Fresh	3.75	59.54	6.88	12.84
Earthen pot T_1_	T_1_a	2.30 −(1.45)	51.79 −(7.75)	6.51 −(0.37)	8.66 −(4.18)
	T_1_b	2.20 −(0.10)	48.93 −(2.86)	0.131 −(6.38)	7.340 −(1.32)
Plastic jar T_2_	T_2_a	2.68	48.36	1.070	9.480
	T_2_b	2.27 −(0.41)	38.56 −(9.8)	0.079 −(0.991)	8.520 −(0.96)
Polythene bag T_3_	T_3_a	1.52	55.77	2.830	9.590
	T_3_b	1.37 −(0.15)	48.98 −(6.79)	0.091 −(2.739)	8.410 −(1.18)
Jute bag T_4_	T_4_a	3.59	50.47	0.75	10.71
	T_4_b	2.34 −(1.25)	48.91 −(1.56)	0.001 −(0.749)	9.040 −(1.67)

Figures in parentheses are the changes in values, a. first year sample, b. second year sample.

#### After 24 months

Twenty four months after the storage of guggul in different containers, the trend of CHNS continued to remain the same, except that there was a decline in percent of all the four elements in all samples stored irrespective of the containers. Guggul samples stored in the jute bags had highest values of N (2.34%) and H (9.04%) while lowest S (0.001%). It indicates the lowest decline in the value of N (37.6%) and H (29.59%) was in jute bag after 24 months of storage in comparison to the fresh guggul samples. However, the highest decline in N was noticed in polythene bag, C in plastic jar, S in jute bag and H in earthen pot in comparison to the fresh guggul samples. The S content was highest (0.13%) in the guggul samples in earthen pots and lowest (0.001%) in jute bags (Table 1).

The estimation of element contents of a consumable sample is useful to know its nutritional value for end users. Carbon, hydrogen, nitrogen and sulphur are important due to their role in enzymatic reactions and protein synthesis^36,37^. Nitrogen and sulphur are good source of nutraceuticals^38,39^. Carbon plays a key role in pH regulation in different parts of human body^40^. Therefore, the presence and quality of these four elements in a natural product has significance in terms of its physico-chemical characteristics.

### FT-IR analysis

The chemical or functional compounds in the sample were investigated using FT-IR spectroscopy—the most appropriate non-destructive spectroscopic method. Here the materials are not subjected to thermal or mechanical energy during sample preparation. FT-IR is widely used in the analysis of pharmaceutical solids, preventing solid-state transformations^41^. Jantasee et al.^42^ and Cozzolino^43^, relied on FT-IR for determination and quantification of functional compounds in plants tissues. Interestingly, Park et al.^44^ used FT-IR to analyze the bioactive indicators of kiwi fruits. FT-IR was used by Lu et al.^45^ to detect total phenols as well as anti-oxidants in garlic and elephant garlic. Gorinstein et al.^46^ also relied on FT-IR for identification of bio-active compounds in mango, avocado and durian. Lam et al.^47^ did it for grapes, blueberry and blackberry. These studies demonstrated that FT-IR is a rapid, reliable and accurate analytical method for determining functional compounds even in natural products such as guggul gum. FT-IR spectroscopy allows for the qualitative identification of organic compounds by identifying the characteristic vibrational mode of each molecular group in the form of bands in the infrared spectrum at a specific frequency, which is more influenced by the contiguous functional groups^48^.

**Figure 1:**
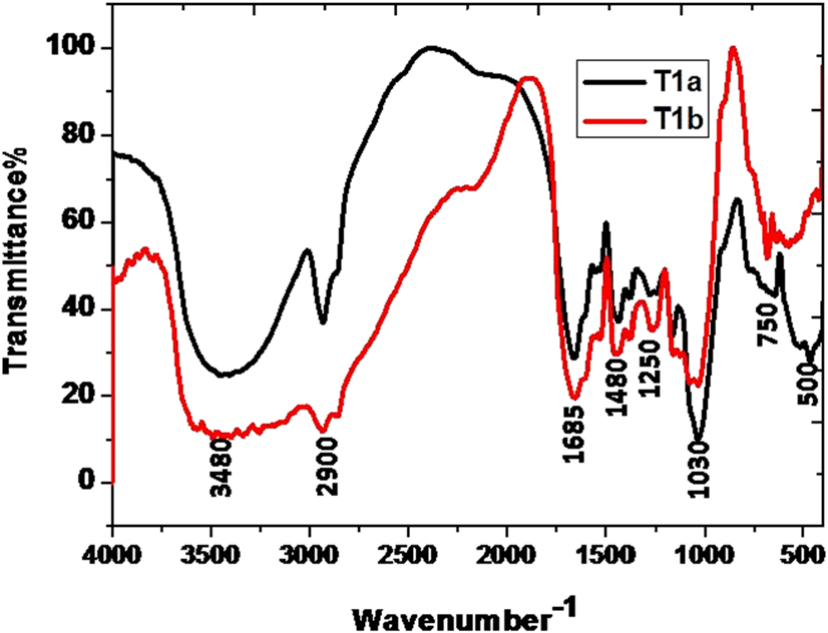
Comparison of FT-IR spectrum of guggul stored in Eathen pots.

**Figure 2:**
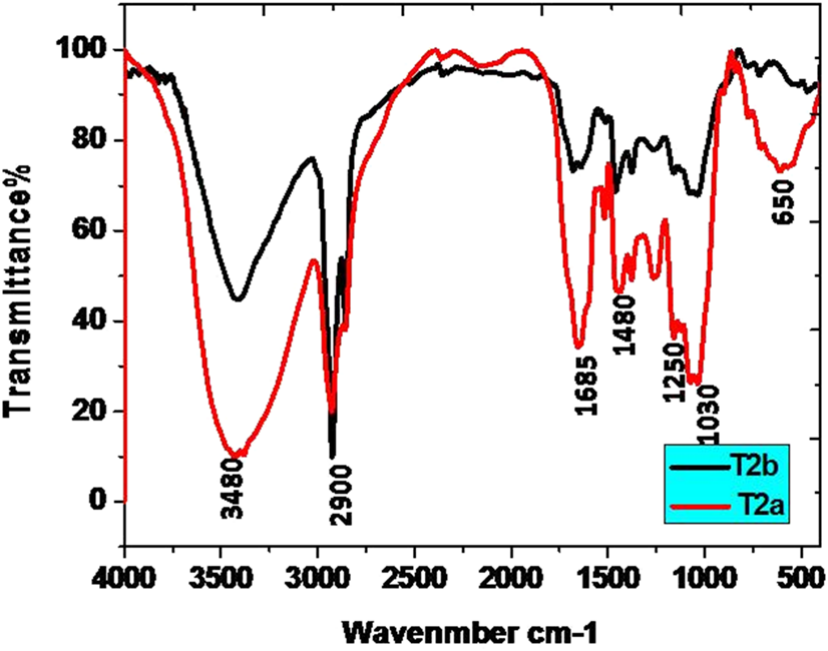
Comparison of FT-IR spectrum of guggul stored in Plastic jars.

**Figure 3:**
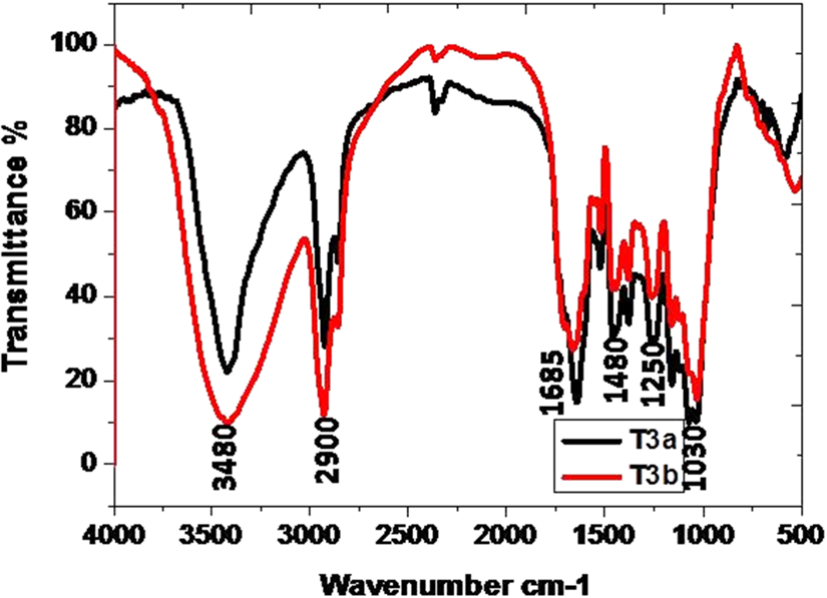
Comparison of FT-IR spectrum of guggul stored in Polythene bags.

**Figure 4:**
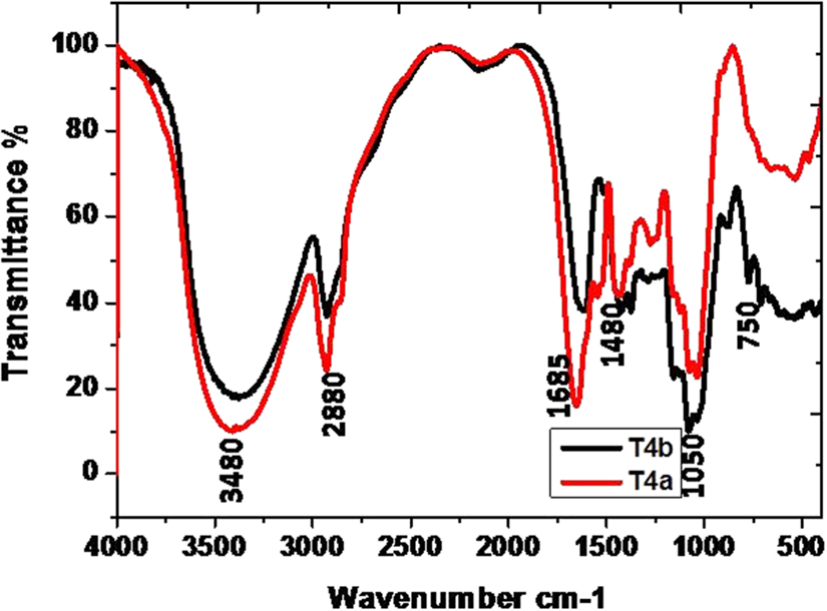
Comparison of FT-IR spectrum of guggul stored in Jute bags.

The data of the FT-IR analysis revealed the presence of a large number of functional groups in all the samples of guggul in the present study. The broad band (BB) of FT-IR spectra (Figs. 1, 2, 3, 4) at higher frequencies, 3500–3200 cm^−1^, is related to the intermolecular hydrogen-bonded O–H stretching vibration of water (H-OH), hydroperoxides (ROOH), and their break-down products, namely alcoholic compounds (ROH) as well as flavonoids. The absorption zone of C–H stretching vibration of methylene and terminal methyl groups of fatty acid chains is known at 3025–2850 cm^−1^ BB region. The presence of C=O groups in the 1630–1695 cm^−1^ BB region indicated the availability of amide groups, carboxylic acids, and derivatives. The BB in the range of 1350–1470 cm^−1^ revealed the presence of CH_2_ and CH_3_ deformation and 1300–1000 cm^−1^ is coupled with the stretching vibrations of C–O ester groups. Generally, the olefinic C–H out-of-plane twisting bands are below 1000 cm^−1^^49^. There were prominent peaks in the region 1307–1025 cm^−1^. Here, the presence of ether, esters, and carboxylic acids, are indicative of a wide range of metabolites, including tannins, flavonoids and anthraquinones, among others^50^.

#### Fresh guggul

FT-IR of fresh guggul had distinct vibrational frequency peaks in the region ~ 3500 cm^−1^, which is assigned to $\upnu$(OH). This may be attributed to higher moisture content and flavonoids. Alkyl stretching frequency at 2850–2930 cm^−1^ $\upnu$(CH_3_) confirms the existence of different terpenoids, phytosteroids such as guggulsterones. Frequency of 1500–1700 cm^−1^ is may be due to the occurrence of α helix protein. The peaks at the region 1033–1164 cm^−1^ indicated the presence of essential linear molecule (glucomannan) in guggul. The differing water-soluble polysaccharides in the graph indicates peaks of absorption representative of polysaccharides with bands at 1033–1164 cm^−1^ and for OH groups at ~ 3500 cm^−1^^51^. The stretching vibrations of OH caused by inter and intra molecular hydrogen bonding indicated by the intense peak at 3383 cm^−1^^52^. The absorption bands at 2850–2930 cm^−1^, according to Li et al.^53^, correspond to CH absorptions (CH, CH_2_ and CH_3_ stretching and bending vibrations). The C = O group is indicated by the signals at 1412 cm^−1^^54^.

#### After 12 months of storage

The FT-IR analysis of guggul samples after 12 months of storage in four different containers viz. earthen pot, plastic jar, polythene bag and jute bag are described here. Guggul sample stored in Earthen pots had the BB at higher frequencies i.e., 3500–3200 cm^−1^, which may be related to.

i. the intermolecular hydrogen-bonded O–H stretching vibration of water (H-OH),
ii. hydroperoxides (ROOH) and
iii. their break-down products,
iv. alcoholic compounds(ROH) and
v. flavonoids

The narrow band of spectra at 3500–3200 cm^−1^ obtained in guggul samples stored in plastic jars, polythene bags and jute bags may be due to rapid loss of moisture as well as early breakdown of alcoholic compounds present in these samples.

The zone of C–H stretching vibration of methylene and terminal methyl groups of fatty acid chains is at 3025–2850 cm^−1^. The intensive bands at 2934 cm^−1^, 2925 cm^−1^, 2925 cm^−1^, and 2927 cm^−1^ were present in the guggul sample of earthen pot, plastic jar, polythene bag, and jute bag respectively. The FT-IR spectrum of the guggul sample of plastic jar and polythene bag had other bands at 2855 cm^−1^ and 2856 cm^−1^ respectively. Surprisingly these were absent in the spectra of samples stored in earthen pot and jute bag, pointing to the possible breakdown of some aromatic compounds in guggul samples stored in plastic jar and polythene bags. No peak at this spectrum interval in the sample stored in jute bags indicates the total loss of aromatic compounds. Porous nature of the jute bags may have contributed to this loss.

The BB at 1655 cm^−1^, 1681 cm^−1^, 1640 cm^−1^ and 1617 cm^−1^ are linked to the C = O stretching absorption of the triglycerides ester linkages. Among all storage guggul samples, the sample stored in plastic jars had a very short peak at this frequency, indicating loss of some triglycerides. The temperature in air tight (plastic) containers usually remains higher than those where gaseous exchange takes place. The possible reason for this loss may be due to higher temperature in air tight plastic jars in comparison to the other storage system. The BB at 1377 cm^−1^, 1376 cm^−1^, 1375 cm^−1^ and 1375 cm^−1^ are related to the symmetrical C–H bending vibrations of -CH_3_ groups in earthen pot, plastic jar, polythene bag and jute bag respectively, while the BB at 1436 cm^−1^, 1457 cm^−1^, 1454 cm^−1^ and 1425 cm^−1^ in earthen pot, plastic jar, polythene bag and jute bag respectively are related the asymmetrical bending vibrations that overlaps the C–H bending vibrations of methylene groups. The BB in the range of 1300–1000 cm^−1^ are associated with the stretching vibrations of C–O ester groups. Usually the olefinic C–H out-of-plane bending vibration bands are at lower than 1000 cm^−1^ frequencies.

Further, the guggul samples stored in plastic jars had very narrow or no peaks at different frequencies. Broad spectra at all these frequencies, indicates that earthen pot is a better storage container, as it maintains all important organic compounds that were present in the fresh guggul samples. The results revealed the superiority of earthen pot storage system for the fresh guggul samples among all four storage systems tested.

#### After 24 months of storage

Fresh samples after its storage for 24 months were subjected to FT-IR analysis. The data of 12 months (a) and 24 months (b) old stored samples of guggul in different containers were compared.

The spectra of both of the samples stored in earthen pots were compared. The BB of 3500–3200 cm^−1^ was observed only in the spectra of guggul sample stored for 12 months. This indicates the presence of higher moisture content and alcoholic compounds but the absence of these BB in guggul sample stored for 24 months demonstrates the loss of both moisture and alcoholic compounds. The 3025–2850 cm^−1^ region represents C–H stretching vibration of methylene and terminal methyl groups of fatty acid chains. The intensive band in guggul sample stored for 12 months in earthen pot present at 2934 cm^−1^ was found to be shorter and absent in guggul sample stored for 24 months. It is again an indication of the absence of some CH_3_ group containing compounds after 24 months of storage in earthen pot. The peaks of other BB, were found to be present in guggul sample stored for 24 months and also in those stored for 12 months in earthen pot.

All the intensive bands present in the guggul sample stored for 12 months in plastic jar, were absent in sample stored for 24 months in plastic jar indicating complete loss of bio-active components.

Unlike that observed in plastic jar, guggul samples stored in Polythene bags of both years had presence of all organic groups in 24 months old stored sample as those in 12 months old stored sample.

BB spectra of the guggul samples stored in Jute bags were found to be present in the same intensity in the 24 months old stored sample as those in 12 months old stored sample except the intensive band at 2927.29 cm^−1^ intervals. This reveals the loss of many aromatic compounds that were present in guggul sample stored for 12 months in jute bag. The short band found at 1617 cm^−1^ was an evidence of the loss of triglycerides ester linkages after 24 months of storage.

The data obtained after FT-IR analysis also revealed the superiority of earthen pots as a storage container among all the storage containers used in the present study. Irrespective of the storage containers, the duration of storage results in qualitative loss of bio-active groups in guggul gum. Ahmad et al.^55^ also used FT-IR to detect the presence of functional groups in guggul. IR spectra of guggul revealed that the gum oleo-resin of *C. wightii* contains several functional groups such as amine, alkanes, lipid, ionic phosphate, polyester overlapping, -helix, modes (–C–H), –C–O–C, and so on. They also found terpenoid, flavonoid, saponin, tannin, protein, alkaloid, glycosides and steroid, among other secondary metabolites. Phytochemical experiments on methanol and ethyl acetate extracts validated these findings. These results revealed the presence of numerous secondary metabolites. The mentioned above compounds also have been identified with their therapeutic values^7,56^. FT-IR spectral analysis of the leaves, stems, and roots of *Eclipta alba* and *E. prostrate* has been done for determination of functional groups of carboxylic acids, amines, amides, sulphur derivatives, polysaccharides, nitrates, chlorates, and carbohydrate^57^. Presence of these functional groups differentiates herbal from non-herbal plants. Kumar et al.^58^ identified protein, oil, lipids, phenolic chemicals, flavonoids, saponins, tannins, and glucose as main functional groups after the FT-IR analysis of methanolic and aqueous leaf extracts of *Bauhinia racemosa*. Perumal et al.^59^ used FTIR to examine the functional group components of amino acids, amides, amines, carboxylic acid, carbonyl compounds, organic hydrocarbons, and halogens in ethanolic extracts of *Ichnocarpus frutescens*.

With seasonal change, Ali et al.^60^ found a decrease in aliphatic and polysaccharide components through FT-IR. Lv et al.^61^ also used this tool to investigate the water extractable organic matter removed from the vermicomposting process of cattle manure. The total phenolic content and antioxidant activity in lyophilized samples of stored pineapple and under different heat treatments was investigated by Santos et al.^62^ with the use of FT-IR. They found that, thermal treatments was investigated by at 50 °C followed by storage lowered the quantity of phenolic compounds by 21–24 and antioxidant capacity by 20–55 percent respectively. FT-IR was employed to determine the polyphenols in different samples like durian, mango, and avocado by Gorinstein et al.^46^. Thus FT-IR is an effective analytical tool used for comparative phytochemical analysis of fresh and stored/preserved samples by many workers. However, Beltran et al.^63^ (2016) found it difficult to ascertain a straight correlation between the age and the conditions of compounds in a *Pinus* resin sample.

### SEM analysis

#### Fresh guggul

SEM analysis revealed that the morphology of fresh guggul sample was viscid. Scientific evidences reveal that viscidity has a direct relation to the presence of higher moisture content. FTIR spectrums are also indicated the presence of moisture in fresh guggul samples.

#### After 12 months of storage

**Figure 5:**
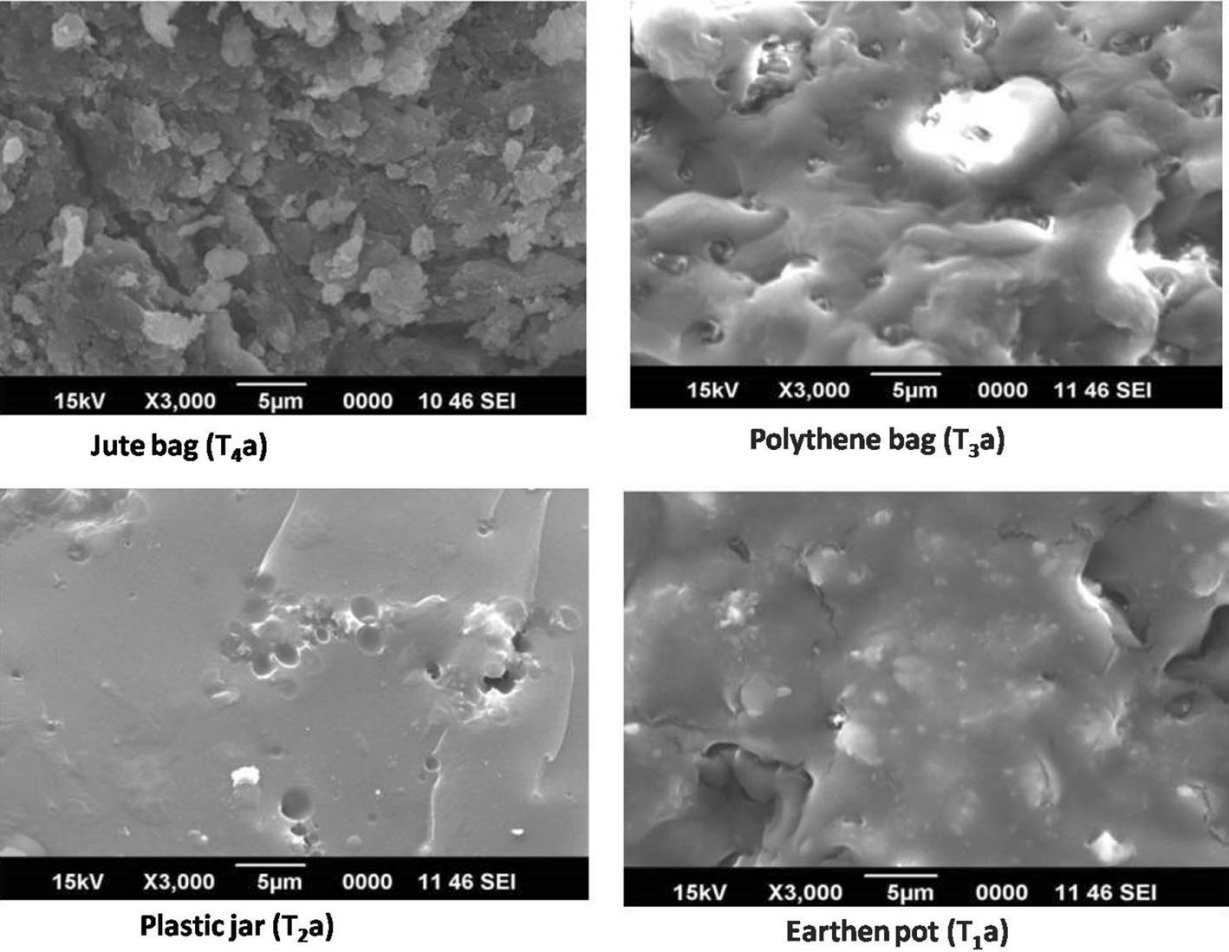
Secondary electron images of gold coated samples of the 12 months old guggul.

Morphology of guggul gum even after 12 months stored in earthen pot was viscid compared to samples stored in other containers (Fig. 5). The inherent character of earthen pot in maintaining cooler inside temperature and relative moisture retention of guggul sample may have contributed to it. FT-IR spectrums also support this. Sample stored in plastic jar and polythene bag, appeared to be rigid but the surface was in a blended form. Sample stored in jute bag was more like flakes or wafers. At higher magnifications this sample appeared free of any blends but as layers or flakes.

#### After 24 months of storage

**Figure 6:**
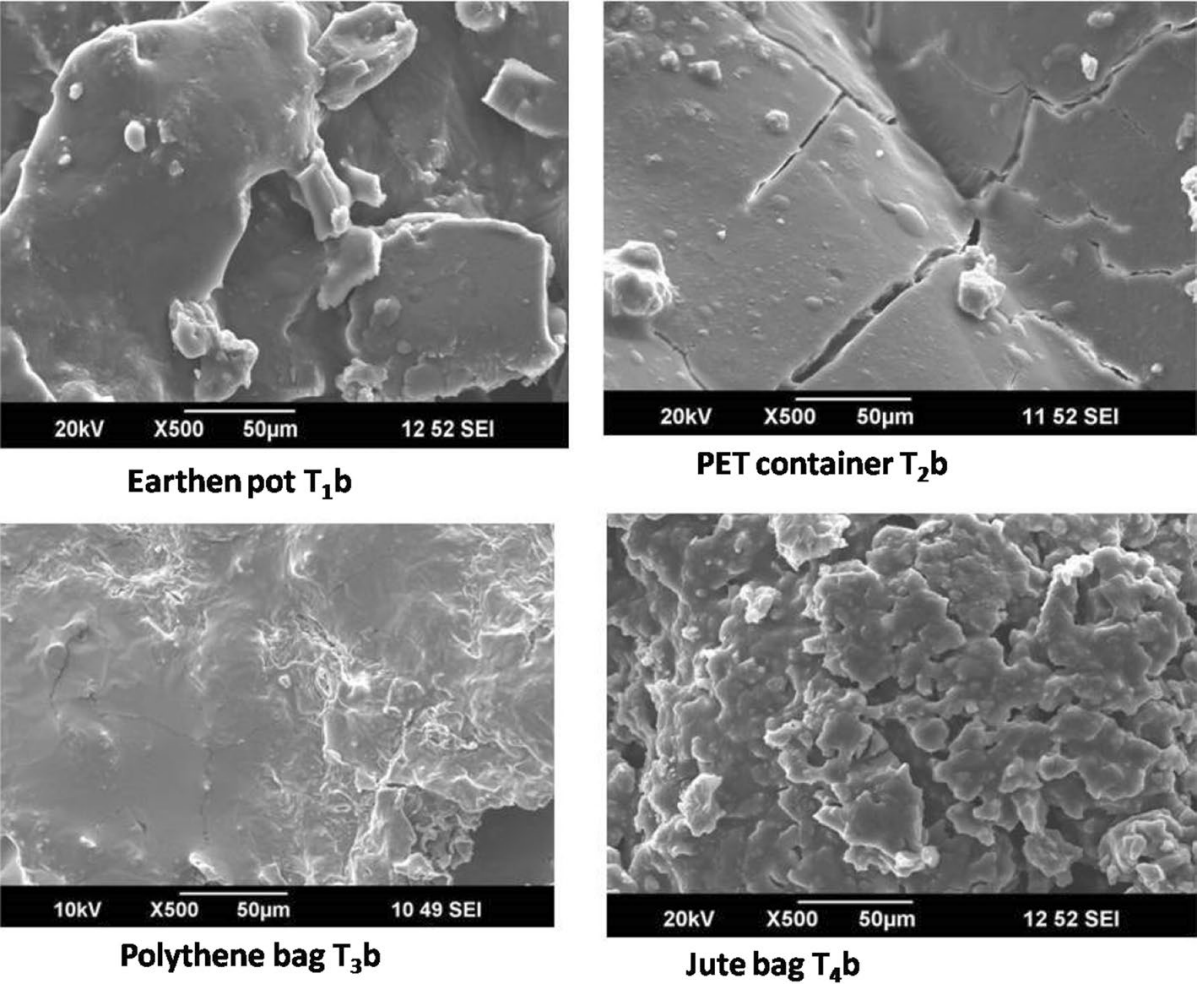
Secondary electron images of gold coated samples of 24 months old stored guggul samples.

Guggul sample stored in earthen pot for 24 months continued with viscid appearance as was in 12 months (Fig. 6). FT-IR spectrums of the sample also support the presence of moisture. In contrast to this, appearance of crack on the surface of the sample stored in plastic jar indicated low moisture content in it. In polythene bag the surface of the guggul appeared in a blended form. The surface morphology of the guggul sample stored in jute bag was more like flakes or wafers. As found in the 12 months stored sample, on a higher magnifications of the sample stored in jute bag for 24 months appeared free of any blends but in layers or flakes. On comparing all the digital images of the 24 months old stored samples of guggul with those stored for 12 months, there was evidence of loss of moisture with the age of storage. Thus, with these evidences, we conclude that moisture content in guggul depends on the container and duration of storage. Among the storage containers, earthen pots appeared to be the best even for 24 months of storage of guggul samples. The present of SEM findings further confirms that guggul samples stored in different containers and duration not only differs in morphologies but also in physical features. This crucial information recorded of guggul gum (natural products) may be immense value in pharmaceutical industry, as it is likely to affect as well as alter their processing during drug formulation^64^ and production.

Morphological or surface textural studies have been done by many workers in the past on drying affect diverse fruits, vegetables, and other food materials^65,66^. Pimpaporn et al.^67^ through SEM demonstrated that potato chips dried at 80° and 90 °C had more consistent pore size and pore distribution than chips dried at 70 °C. The latter had more broad surface shrinkage. When Fang et al.^68^ carried out the milk spray drying under different drying temperatures; they found that lower drying temperatures resulted in relatively uniform size and shape with smooth particle surfaces, against higher drying temperatures where there was variations in size and wrinkled particle surfaces. Thuwapanichayanan et al.^69^ investigated the effect of drying temperatures on moisture diffusivity and qualitative parameters of dried banana slices, including volatile compounds, shrinkage, colour, texture and microstructure. Vega-Gálvez et al.^65^ did a detail study on the effects of various pretreatments on the microstructure of Aloe vera gel during convective drying at 70 °C, including high hydrostatic pressure, blanching, enzymatic, and microwaves. These studies confirm use of SEM micrographs as novel approach to investigate, interpret and even anticipate the rehydration or textural aspects of dried items. As a, similar important investigative report on guggul gum was lacking.

### TGA

The temperature behaviour of medicinally essential compounds is critical as it can reveal valuable information on formulation stability. It may also helps to identify incompatibility between the medicine and the excipient^70^. The temperature of initial (TID) and maximum decomposition (TMD) of a material is important information obtained by TGA. The curves of TGA illustrate weight loss of the sample as a function of temperature. Needless to mention the role of thermal steadiness of bio-active compounds is a noteworthy base for their preference and applying in the pharmaceutical applications. During the present study, the fresh guggul sample exhibited an endothermic phase transition near 160 °C. This is the TID where material initiates decomposition. The guggul sample stored for 24 months in earthen pots (T_1_b) had an TID at about 100 °C against 140 °C in 12 months old sample (T_1_a). This may be due to presence of high moisture content in guggul sample stored in earthen pot for 12 months (T_1_a) as compared to sample in earthen pot for 24 months (T_1_b). Maximum rate of decomposition temperature (TMD) for fresh guggul was nearly 300 °C indicated that the regime when the maximum component in the material is decomposing. The TMD of guggul sample stored in earthen pot for 24 months (T_1_b) was near 280 °C. The TID of the 24 months old guggul samples stored in plastic jars (T_2_b) was at about 100 °C whereas for 12 months old sample (T_2_a) it was at near 120 °C. The TMD of T_2_a was at near 280 °C while for T_2_b it was near 210 °C. The TID of the 24 months old stored guggul sample in polythene bag (T_3_b) was at 100 °C while that 12 months old guggul sample (T_3_a) was at near 140 °C. This indicates presence of less moisture content in the 24 months old samples. The TMD at low temperature indicates loss of other organic compounds in the sample. Similar trend was found during comparative analysis of TGA graphs for the guggul sample stored in jute bag (T_4_). The TGA clearly indicated that with the age of storage of guggul the TID and TMD occurs at lower temperature. It also depends on the container in which the guggul was stored. When the TMD is compared among the guggul stored in different containers and duration, it revealed valuable information (Table 2) on the safe operational temperature of guggul while processing or value addition with minimum loss of components. No literature is available on this aspect of guggul gum. Higher TID and TMD may be advantageous in processing, value addition of any pharmaceutical drug or complete products. In this context, our result again confirms that guggul gum may be stored in earthen pots.

**Table 2:** Decomposition temperatures of fresh and stored guggul samples.

Guggul	Temperature of decomposition °C					
	Initial		Difference °C	Maximum		Difference °C
Fresh	160°C					
Storage Container	12 months	24 months		12 months	24 months	
Earthen pot T_1_	140 °C	100 °C	40	280 °C	275 °C	5
Plastic jar T_2_	120 °C	100 °C	20	280 °C	210 °C	70
Polythene bag T_3_	140 °C	100 °C	40	140 °C	100 °C	40
Jute bag T_4_	110 °C	95 °C	15	300 °C	290 °C	10

### X-ray diffraction analysis

PXRD analysis was performed to identify the crystallinity of samples. The peaks indicate the presence of high moisture content in the sample.

#### After 12 months storage

**Figure 7:**
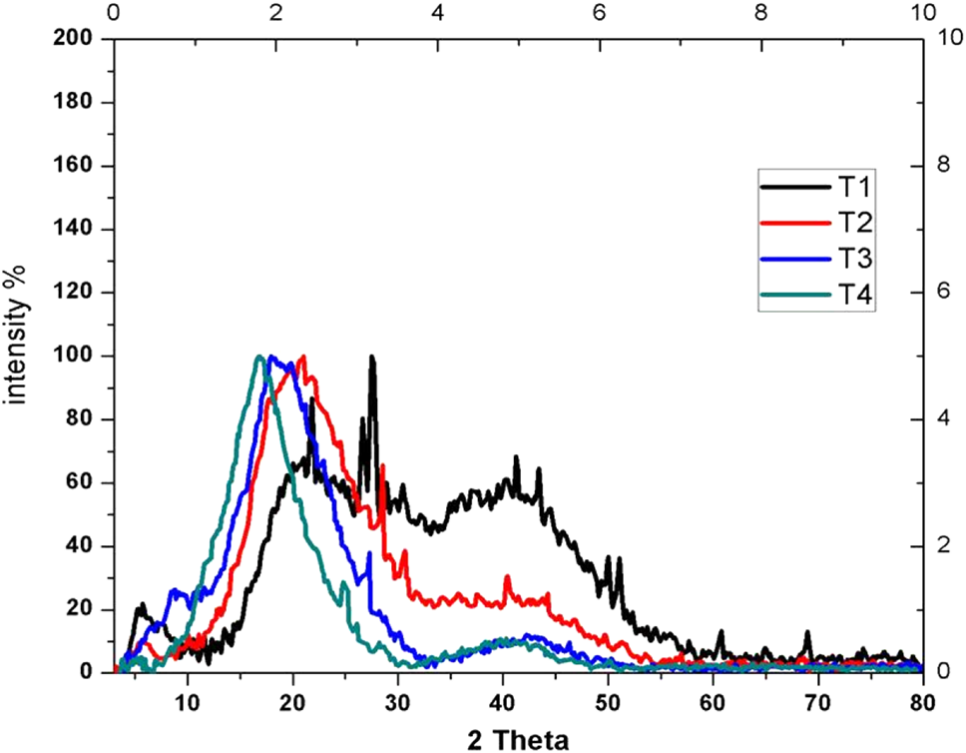
Crystalline peaks of the samples stored in different conditions.

Diffractogram of the guggul gum sample stored in earthen pot (T_1_a) exhibited higher degree of crystallinity (Fig. 7). The sharp crystalline peaks at various 2 *theta* values were conspicuous in the sample stored in earthen pot (T_1_a) while samples stored in plastic jar (T_2_a), polythene bag (T_3_a) and jute bag (T_4_a) did not exhibit such a crystalline nature. Nevertheless the later three exhibited less intense peaks at 25 °C. The guggul sample stored in jute bag had the least intense peak at this angle. The sample stored in plastic jar (T_2_a) shared many crystalline peaks similar to the sample stored in earthen pot (T_1_a). However the former lacked the intensity (Fig. 7) of the latter sample.

The sharp crystalline peaks in sample stored in earthen pot indicated the presence of crystallites in this natural polymer. This may not have been reported earlier. The morphology (SEM analysis) of sample stored in earthen pot and in plastic jar appeared like a blend. The guggul sample stored in polythene bag to a certain extent compared to wafers as sample stored in jute bag. Ability of earthen pot to preserve the crystallite morphology can add value to guggul in processing and product formulations. Changes of crystallinity may be due to the degree of crosslinking in the samples, controlled by hydroxyl groups existing in the guggul samples^71^. The decrease in the degree of crystallinity falls in the order from sample stored in earthen pot to those stored in jute bag. Guggul being a natural compound; it is difficult to identify its components specifically using X-Ray analysis.

#### After 24 months storage

**Figure 8:**
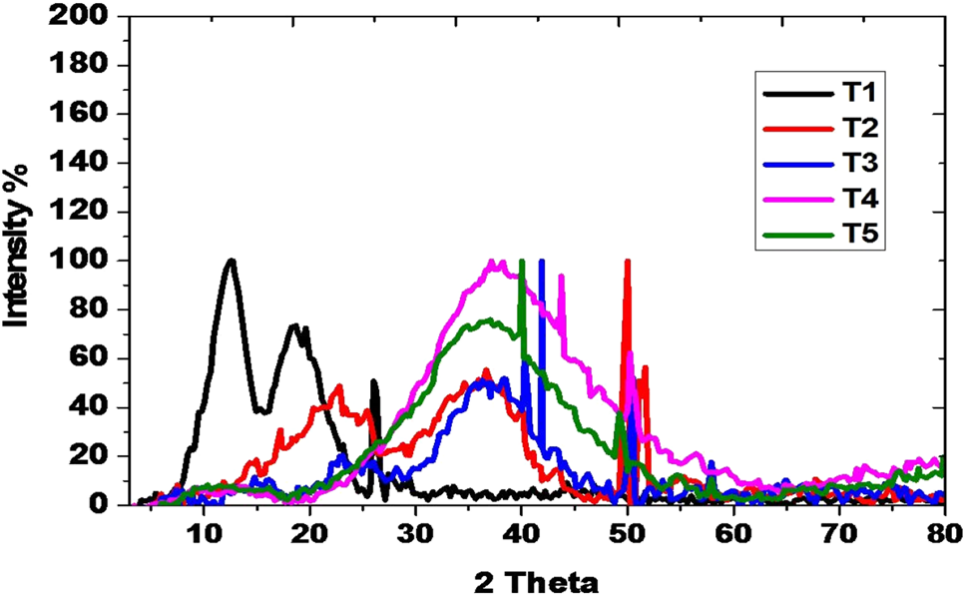
Crystalline peaks of the guggle samples stored for 24 months in different containers.

Diffractogram of the guggul gum sample stored for 24 months in earthen pots exhibited lesser degree of crystallinity in comparison to that in 12 month stored sample (Fig. 8). Samples stored in plastic jars, polythene bags, jute bags and high density polyethelene bag did not exhibit such a crystalline nature and had less intense peaks. The guggul sample stored in jute bags had the least intense peak at this angle. The sample stored in plastic jars though shared many peaks similar to that stored in earthen pots but were less intense indicating of amorphousness of the sample.

The guggul stored in earthen pots exhibited crystallinity, while those stored in other containers were amorphous. The crystalline state is more stable than the amorphous one. The components of crystalline solids are arranged in regular ordered arrays and held together by consistent intermolecular forces, whereas it was not in the components of amorphous solids. The low aqueous solubility of particles may be due to its crystallinity. Therefore, the amorphization that occurs when drugs are prepared as solid dispersions has a significant dissolution rate of the drug^72^.

## Conclusions

The unique absorptions of all samples of guggul gum oleo-resin from fresh and stored in different containers and period were revealed by FTIR spectra and TGA curves. Presence of diverse bio-active and functional groups in guggul samples had a significant impact on the rigidity of the materials, which is consistent with the results of XRD analysis and mechanical properties. There is an evidence of varying degree of crystallinity of guggul samples stored in various containers and storage period. Samples in earthen pots had pronounced crystalline nature of guggul but degree of crystallinity decline with the age of storage. Thus all the physico-chemical analysis (CHNS, FTIR, SEM, TGA and X-Ray diffraction) strongly suggests guggul gum may be stored in earthen container. However the length of storage reduces qualitative parameters of guggul. The ayurvedic and cosmetic industry at large may store guggul in earthen containers to maintain the therapeutic quality of guggul.

## Data Availability

All data generated or analysed during this study are included in this published article.
